# *Parastrongylus cantonensis* in a Nonhuman Primate, Florida

**DOI:** 10.3201/eid1012.040319

**Published:** 2004-12

**Authors:** Michael S. Duffy, Christine L. Miller, J. Michael Kinsella, Alexander de Lahunta

**Affiliations:** * Cornell University, Ithaca, New York, USA;; † Miami Metrozoo, Miami, Florida, USA;; ‡ HelmWest Laboratory, Missoula, Montana, USA

**Keywords:** Parastrongylus (= Angiostrongylus) cantonensis, central nervous system, parasitic infections, nematode, neurologic diseases, eosinophilic meningitis, zoonoses, primates, Florida, dispatch

## Abstract

*Parastrongylus* (= *Angiostrongylus*) *cantonensis* is a parasitic nematode of Norway rats throughout tropical regions. This parasite is neurotropic and causes disease and death in humans and other mammals. We report the first identification of *P. cantonensis* as the cause of a debilitating neurologic disease in a captive primate in Florida.

## The Case

On July 28, 2003, an acute and disabling neurologic disorder developed in a captive 49-year-old male white-handed gibbon (*Hylobates lar*) from the Miami Metrozoo in Florida. The gibbon was born in the wild but had been in captivity in Florida, USA since 1963. The onset of severe quadriparesis occurred overnight, without signs of prior illness. In addition to extreme weakness of all limbs and the inability to support his body, the gibbon had a slight lip droop but was able to swallow and had no other detectable cranial nerve signs. The gibbon was behaviorally depressed but responsive and aware of his surroundings. Blood analyses and thoracic radiographs did not show a cause for the clinical disease, and blood eosinophil count was not elevated. The animal did not show improvement after 48 hours and was euthanized by intravenous injection of Euthasol (Delmarva Laboratories Inc., Midlothian, VA). On postmortem examination, chronic renal disease and moderate cardiac fibrosis and endocardiosis were observed, but these conditions were considered to be age-related. Gross abnormalities were absent on external surfaces of the central nervous system (CNS). Tissues of the CNS were preserved in 10% formalin.

**Figure 1:**
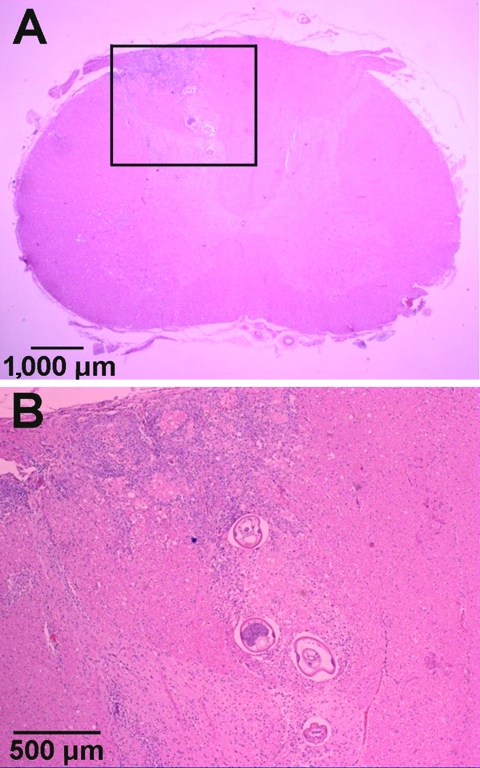
Hematoxylin and eosin–stained sections of *Parastrongylus cantonensis* in the parenchyma of the cervical spinal cord of a gibbon (*Hylobates lar*) from Florida (A). Enlarged image of inset from panel A (B).

Tissue samples from regions of the CNS were embedded in paraffin by using standard procedures. Tissue sections were cut (6 μm) and stained with hematoxylin and eosin. In the transverse sections, the only gross lesion observed was a thin, white discolored area in the left dorsal funiculus in the cranial cervical spinal cord segments. Microscopic examination showed necrosis and inflammation located primarily in the dorsal gray columns throughout the cervical spinal cord. This lesion was bilateral with some involvement of the adjacent funiculi. The areas of inflammation included numerous eosinophils and transverse sections of one or more parasitic nematodes (Figure 1). A few lesions were found in the ventral gray columns. This destructive lesion extended into the caudal medulla. Rostral to this were a few scattered destructive lesions, and a few sections of the parasite were present in the leptomeninges. Wallerian degeneration occurred in the dorsal funiculus where the white discoloration was observed on gross examination.

**Figure 2:**
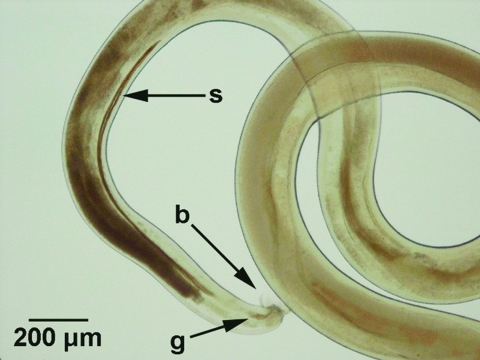
Morphologic features of a male nematode recovered from the central nervous system of a gibbon (*Hylobates lar*). The characteristics used for specific identification of *Parastrongylus cantonensis* were the presence of a bursa (b), a gubernaculum (g), and the size of spicules (s).

Preserved tissues of the CNS were examined for nematodes at 4x magnification with a dissecting microscope (Zeiss, Micro-Med Instruments, Inc., Walden, NY). Four nematodes were recovered from the meninges of the brain and spinal cord. One intact male nematode and additional pieces of a nematode were recovered from the subarachnoid space of the cervical spinal cord. A second intact male nematode was recovered from the subarachnoid space of the cerebellum, and additional pieces of a male nematode were recovered from the subarachnoid space of the cerebrum. The partial nematodes had damaged anterior and posterior ends. Nematodes were examined at 40–400x magnification, and digital images of specimens were captured (Olympus Model DP12, Olympus Optical Co., Ltd., Tokyo, Japan). Corresponding images of a stage micrometer allowed the sizes of morphologic characteristics to be determined. A key morphologic feature of the three male nematodes was the presence of a well-developed bursa (Figure 2). This feature indicated their classification within the order Strongylida. Within this group, members of the superfamilies Diaphanocephaloidea, Ancylostomatoidea, and Strongyloidea were excluded based on morphologic features of the anterior end. Members of the Trichostrongyloidea were also excluded on the basis of the absence of a cephalic vesicle and longitudinal cuticular ridges. In addition, with the exception of the family Dictyocaulidae, all Trichostrongyloids are restricted to the intestinal tract. Furthermore, no strongylid nematodes from the aforementioned superfamilies have been reported from the CNS. The nematodes from the gibbon CNS were thus classified as members of the superfamily Metastrongyloidea.

Sprent (*1*) and Anderson (*2*) documented extensively the nematodes reported from the CNS of mammals. Within the Metastrongyloidea, these include *Parelaphostrongylus* spp., *Elaphostrongylus* spp., *Skrjabingylus* spp., *Gurltia paralysans*, and *Parastrongylus* spp. Of those nematodes, only *Parastrongylus* spp. have been reported previously from the CNS of primates. As such, the causative agent was presumed to be a species of the genus *Parastrongylus*. This genus comprises *P. cantonensis*, *P. malaysiensis*, *P. mackerrasae*, *P. sandarsae*, *P. siamensis*, *P. costaricensis*, *P. dujardini*, *P. schmidti*, *P. tateronae*, *P. ryjikovi*, *P. sciuri*, and *P. petrowi* (*3*). Of these species, *P. cantonensis*, *P. malaysiensis*, and *P. costaricensis* have been reported previously from primates.

Spicule measurements (1,140–1,180 μm) of nematodes from the gibbon (Figure 2) correlated well with measurements reported for *P. cantonensis*. With the exception of *P. malaysiensis*, *P. cantonensis* is distinguished from all other *Parastrongylus* spp. based on spicules >1,000 μm. Spicules from *P. malaysiensis* are on average 940 μm (800–1,200 μm). *P. cantonensis* and *P. malaysiensis* are distinguished on the basis of the morphology of bursal rays from male nematodes (*4*). The bursal rays of nematodes recovered from the gibbon were consistent with those of *P. cantonensis*. Based on a combination of morphologic features, spicule measurements, host species, and location within the host, we concluded that the infecting nematodes were *P. cantonensis*. The male specimens of *P. cantonensis* were deposited in the U.S. National Parasite Collection (Beltsville, MD) under accession number 94698.

## Conclusions

*P. cantonensis* are parasitic nematodes that reside in the cardiopulmonary system of their rat (*Rattus* spp.) definitive hosts. The parasite is common in Southeast Asia and the South Pacific and has been reported in Madagascar, Japan, Egypt, and India (*5*). Reports also document *P. cantonensis* from the Western Hemisphere in Cuba, Puerto Rico, Dominican Republic, Bahamas, Jamaica, and Haiti (*6**,**7*). From the mainland United States, *P. cantonensis* is known only in Louisiana (*8*) and Mississippi (*9*). Naturally acquired infections in these two areas resulted in neurologic disease in a horse (*9*), in wildlife species (*10*), and in both human (*11*) and nonhuman primates (*10**,**12*). Onset of neurologic signs in humans occur 1–45 days after infection (*13**,**14*). This feature suggests that the captive gibbon in this report likely acquired the infection shortly before onset of disease. The advanced age of the animal may have been a factor in the severity of the disease. Although deaths have been reported in human adults, infections typically result in transient neurologic debilitation (*14*). Conversely, death can occur in 5% of children (*13*). Comparative data are lacking for disease severity in older persons.

Transmission of *P. cantonensis* infection requires first-stage larvae (L1) from rat feces to infect and develop into the infective-stage (L3) within obligate gastropod intermediate hosts. Infection of rats and other mammalian hosts requires ingestion of L3, commonly through ingestion of infected gastropods. However, the L3 of *P. cantonensis* can also emerge in gastropod mucus trails and thereby contaminate surrounding vegetation (*15*). This mode of transmission was reported in a human outbreak of eosinophilic meningitis in Jamaica, where disease was correlated with eating Caesar salad (*16*).

The present distribution of *P. cantonensis* within the United States is unknown outside of Louisiana (*8*) and Mississippi (*9*). The finding of *P. cantonensis* in the captive gibbon in this report suggests that the parasite may be established in Miami, Florida. Since neurologic disease may occur in human and nonhuman primates, dogs, horses, and numerous other species (*9**–**12*), documentation of the present distribution of *P. cantonensis* will prove valuable for monitoring the spread of this zoonotic parasite. *P. cantonensis* have a number of susceptible aquatic (*17*) and terrestrial gastropod intermediate hosts in the eastern United States (*2**,**8*). In combination with the ubiquitous distribution of rats, the probability for spread and establishment of *P. cantonensis* exists within eastern North America.

The dispersal of *P. cantonensis* has, in some instances, been linked to the introduction of the African giant land snail, *Achatina fulica* (*5*). Three specimens of *A. fulica* were introduced into Miami, Florida, from Hawaii in 1966 (*18*). These exotic snails became established but were considered eradicated from Florida by 1975 (*18*). A survey of *A. fulica* in Hawaii in the mid-1960s showed that *P. cantonensis* infection was both highly prevalent and intense (*19*), which suggests that the parasite may have been introduced to Miami with the translocated snails in 1966. *P. cantonensis* was believed not to establish successfully (*18*). However, this event or a similar translocation involving less conspicuous exotic gastropods may have resulted in the inadvertent introduction of *P. cantonensis*. Live *A. fulica* are confiscated routinely from tourists returning to mainland United States from Hawaii, and 75 other exotic gastropod species have been introduced to the United States accidentally, inadvertently, or intentionally (*20*). The introduction of *P. cantonensis* with infected rats from areas where the parasite is endemic would be equally conceivable, as was proposed for its introduction to New Orleans, Louisiana (*8*).

*P. cantonensis* may be established in rat and gastropod populations in Miami. However, given the role of emergent L3 in transmission of infections (*15**,**16*), the L3 involved in the present case may have been acquired from contaminated vegetation supplied commercially from another region. The gibbon in this report was fed a commercial monkey chow and a variety of produce both grown locally and imported from several regions of North, Central, and South America. *P. cantonensis* is not known to be in any of these supply regions. The source of *P. cantonensis* in the gibbon infection remains to be determined. However, *P. cantonensis* was definitely introduced to Miami through the translocation of either infected animals or contaminated plants.

Future investigations aim to conduct parasitologic surveys of rats and gastropods in Miami, Florida, and develop molecular tools for specific identification of *P. cantonensis*. The specific source of infection in the present case report remains unknown. However, our report indicates that infection with *P. cantonensis* should be included as a differential diagnosis for instances of neurologic disease in human and nonhuman primates, as well as in wildlife and veterinary species in the southeastern United States.
